# Deep learning applications for the classification of psychiatric disorders using neuroimaging data: Systematic review and meta-analysis

**DOI:** 10.1016/j.nicl.2021.102584

**Published:** 2021-02-10

**Authors:** Mirjam Quaak, Laurens van de Mortel, Rajat Mani Thomas, Guido van Wingen

**Affiliations:** Amsterdam UMC, University of Amsterdam, Department of Psychiatry, Meibergdreef 5, 1105 AZ Amsterdam, The Netherlands

**Keywords:** Deep learning, Machine learning, Psychiatry, Neuroimaging, Artificial Intelligence

## Abstract

Deep learning (DL) methods have been increasingly applied to neuroimaging data to identify patients with psychiatric and neurological disorders. This review provides an overview of the different DL applications within psychiatry and compares DL model accuracy to standard machine learning (SML). Fifty-three articles were included for qualitative analysis, primarily investigating autism spectrum disorder (ASD; n = 22), schizophrenia (SZ; n = 22) and attention-deficit/hyperactivity disorder (ADHD; n = 9). Thirty-two of the thirty-five studies that directly compared DL to SML reported a higher accuracy for DL. Only sixteen studies could be included in a *meta*-regression to quantitatively compare DL and SML performance. This showed a higher odds ratio for DL models, though the comparison attained significance only for ASD. Our results suggest that deep learning of neuroimaging data is a promising tool for the classification of individual psychiatric patients. However, it is not yet used to its full potential: most studies use pre-engineered features, whereas one of the main advantages of DL is its ability to learn representations of minimally processed data. Our current evaluation is limited by minimal reporting of performance measures to enable quantitative comparisons, and the restriction to ADHD, SZ and ASD as current research focusses on large publicly available datasets. To truly uncover the added value of DL, we need carefully designed comparisons of SML and DL models which are yet rarely performed.

## Highlights

• Deep learning methods are a promising tool for classification of individual psychiatric patients.
• Classification performance is better for schizophrenia than autism spectrum disorders than ADHD.
• Research is mainly focused on large publicly available datasets.
• There is large heterogeneity in choice of preprocessing, features, models and evaluation.
• Studies often lack sufficient performance measures for quantitative comparisons to conventional machine learning methods.

## 1 Introduction

Clinical psychiatry is based on observation and self-report which are inherently subjective. There are no biomarkers available that could enable objective diagnosis or biology-based treatment targeting. Promising approaches for the development of biomarkers include non-invasive neuroimaging techniques such as structural or functional magnetic resonance imaging (MRI) that can capture the structure and function of the healthy and diseased brain. Over the last two decades, many neuroimaging studies have been performed to gain insight in the neural correlates of psychiatric disorders. Most of these studies have compared patients to controls and reported neuroanatomical or neurofunctional differences. This raised hopes of finding imaging biomarkers that could aid the diagnostic process. However, these studies typically relied on mass univariate analysis (group level statistical analysis) and reported group level differences in specific voxels or regions of interest (ROI) in the brain, whereas several psychiatric symptoms are best explained by network-level changes in structure and function rather than specific local alterations (Sheffield and Barch, 2016, Mulders et al., 2012, Kennedy and Courchesne, 2008, Rubinov and Sporns, 2010, Gong et al., 2009).

As the vast amount of data in neuroimaging scans has made it challenging to integrate all the data available, the neuroimaging community has developed a growing interest in machine learning (ML) approaches. ML algorithms are mathematical models that are developed to learn patterns in existing data in order to make predictions on new data. A major advantage of ML techniques is their ability to take inter-regional correlations into account, enabling detection of subtle and spatially distributed effects in the brain (Orrù et al., 2012). Moreover, whereas mass-univariate results explain group differences, ML models allow statistical inference at the level of the individual that could aid individual diagnostic or prognostic decisions (Arbabshirani et al., 2017).

Well-known pattern analysis methods, such as linear discriminant analysis (LDA), logistic regression (LR) and support vector machine (SVM) have been applied to neuroimaging data to detect psychiatric disease with varying degrees of success (Arbabshirani et al., 2017). Classification studies using ML algorithms on highly dimensional neuroimaging data usually require several preprocessing steps involving feature extraction and feature selection to reduce the input dimensions (Lu and Weng, 2007). These procedures require subjective feature choices that raise reproducibility issues (Samper-González et al., 2018).

**Fig. 1:**
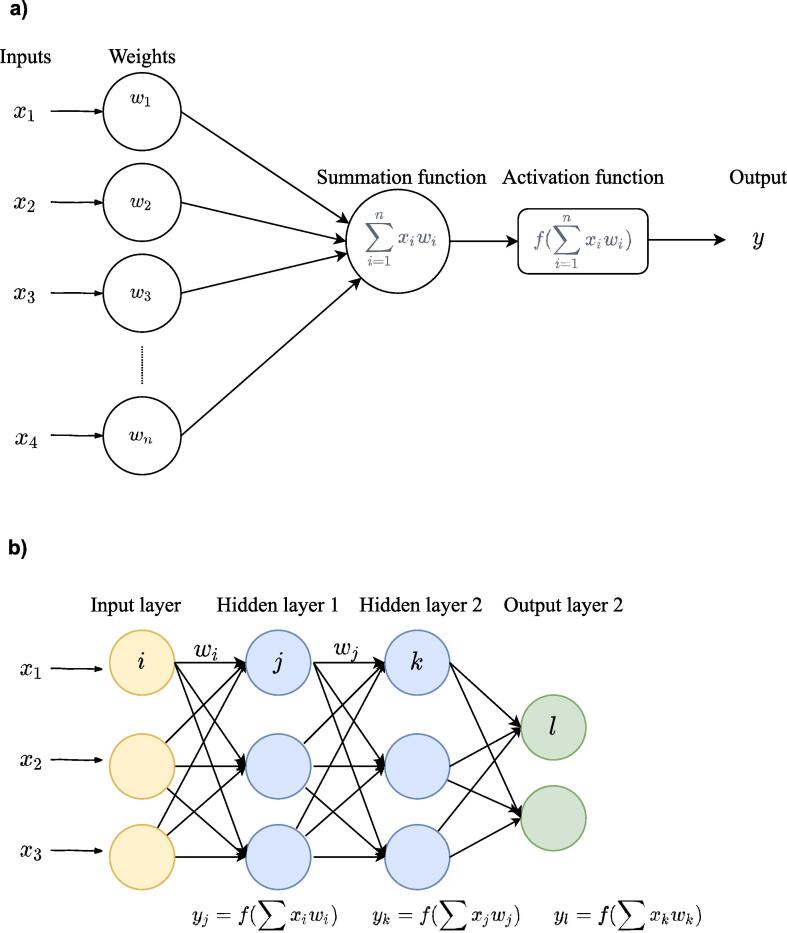
a). An artificial neuron or node. Each input × is associated with a weight w. The sum of all weighted inputs is passed onto a nonlinear activation function f that leads to an output y. b) An example of a multilayer perceptron. It shows input layer, two hidden layers and an output layer. For each neuron in the first hidden layer, a nonlinear function is applied to the weighted sum of its inputs. The result of this transformation is the input for the consecutive layer.

**Fig. 2:**
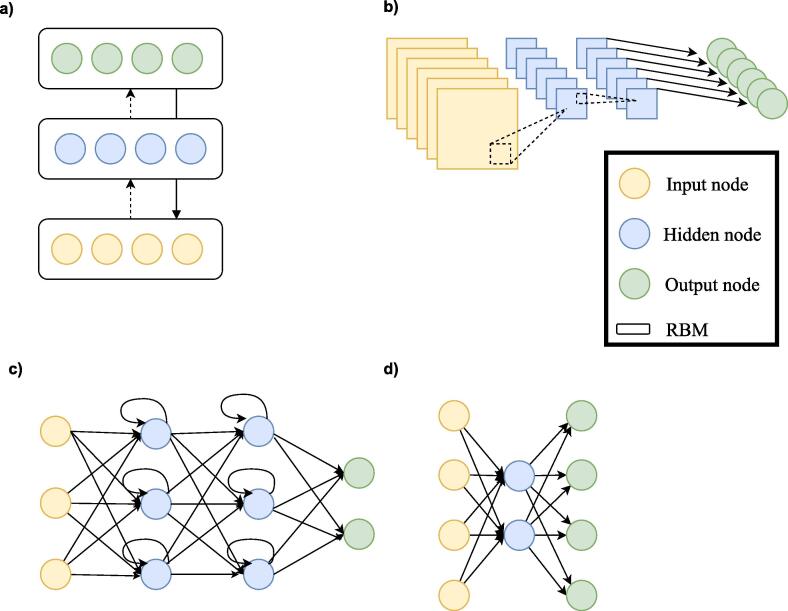
Architectural structures in deep learning. A. Deep Belief Network (DBN). B. Convolutional neural network (CNN). C. Recurrent neural network (RNN). D. Auto Encoder (AE).

After breakthroughs in performance in a large variety of fields, deep learning (DL), a specific class of machine learning algorithms, has found its way into the neuroimaging community. DL models are hierarchical models that achieve increasingly higher levels of abstraction and complexity by stacking consecutive nonlinear transformations (see Fig. 1, Fig. 2, Box 1 and Vieira et al., 2017 for an introduction). This ability makes DL specifically suitable for neuroimaging applications as psychiatric and neurological disorders are often characterised by complex, subtle and diffuse patterns (Plis, 2014). Moreover, an essential difference between standard machine learning (SML) and DL techniques is that DL enables the learning of optimal feature representation from the raw data, eliminating the need for subjective feature engineering for SML techniques. This results in a more objective and less bias-prone process in DL (Vieira et al., 2017).

**Table:** 

**Box 1**. A short introduction to deep learning
Deep learning is a group of machine leaning methods that tries to learn features from the data by a hierarchical structure of consecutive nonlinear transformations. In the present review, we define a deep learning model as follows: a model is a deep model when it included two or more stacked layers and therefore learns features through a hierarchical learning process. Although deep learning is a subgroup of machine learning, when we refer to machine learning in this review, we refer to shallow machine learning models (such as support vector machines).
The building blocks of deep learning methods are called artificial neurons (see Fig. 1a). The simplest form of an artificial neuron is the single-layer perceptron as proposed by Rosenblatt (1958). The perceptron takes inputs *x* that are multiplied with connection weights *w*. The sum of all weighted inputs is then passed onto a nonlinear activation function such as tanh, sigmoid or rectified linear unit (ReLu). The main idea of the perceptron is to learn the values of the weights *w* in order to make a decision whether the neuron should fire or not.
By stacking several of these neurons, a multi-layer perceptron (MLP) is created (see Fig. 1b). An MLP is organized in layers; an input layer, one or more hidden layer(s) and an output layer. In the input layer, the input data is where the data is entered into the model, the hidden layers learn increasingly abstract features and the output layer assigns a class using the learned features. The type of network determines how these artificial neurons are connected to other neurons. The simplest form of a deep network is the multilayer perceptron (MLP), which is fully connected, meaning that each neuron is connected to all neurons of the previous layer. Each connection is associated with a weight value, reflecting the strength and direction (positive or negative) between two neurons in the network.
During training, the network learns through a gradient descent-based algorithm, that aims to find the optimal weights that lead to a minimal error between predicted and true outputs. The idea behind training with gradient descent is as follows: as training data is fed through the network, the gradient of the loss function is computed with respect to every weight using the chain rule, and the weights are changed using gradient descent.

**Table:** 

**Box 2**. Deep learning architectures
Besides MLPs, there exists a wide variety of deep learning architectures. We will shortly discuss the most common architectures in neuroimaging here (see Fig. 2). For a more elaborate overview of methods see Jo et al., 2019, Vieira et al., 2017
A. Deep belief network (DBN)
Whereas MLPs only have feedforward connections, the DBN has undirected connections between some layers. These undirected layers are called Restricted Boltzmann Machines (RBM) and can be trained both supervised and unsupervised.
B. Convolutional neural network (CNN)
CNNs are mostly used in image recognition. They work by learning ‘convolutions’ or ‘filters’ to detect features. By convolving images, it reduces the data into a form that is easier to process, without losing critical information.
C. Recurrent neural network (RNN)
RNNs do not only contain feedforward connections, but also feedback connections. These feedback connections allow the retainment of information from previous inputs (akin to a form of memory) to affect the current output. The most effective RNNs are gated RNNs such as long short-term memory (LSTM) and networks based on the gated recurrent unit (GRU).
D. Auto Encoder (AE)
AE is an unsupervised learning method that is used to encode the data in a smaller latent representation. They consist of an encoder and decoder part and are trained by making the output value approximate to its input value.

A previous review from 2017 has shown that DL methods have been successfully applied in neuroimaging to classify Alzheimer, ADHD, and to predict disease conversion (Vieira et al., 2017). Since then, the advent of data-sharing initiatives and advances in DL have led to a large increase in DL applications in psychiatry. They show great promise for uncovering reproducible patterns of brain structure and function across larger and heterogeneous datasets (Durstewitz et al., 2019, Bzdok and Meyer-Lindenberg, 2018).

However, there is still a lack of carefully designed comparisons to benchmark SML and DL performance in brain imaging tasks. Like preceding influential technologies, the expectations of future performance of DL in brain imaging might be hyped (Abrol et al., 2020) and it is still unclear to what extent it improves capturing the relationship between brain activity and behaviour. Whereas some studies suggest that DL does not improve prediction of behaviour as compared to simple, linear models (He et al., 2020, Schulz et al., 2019, Guerdan, et al., 2019, others claim that there exists both linear and non-linear relationships between brain connectivity and behaviour and that DL is best capable of fitting both (Bertolero and Bassett, 2020). DL seems to improve classification of brain age and sex prediction (Peng et al., 2021) and DL have been reported to improve AD detection (Vieira et al., 2017, Jo et al., 2019), but whether DL improves classification of psychiatric disease has yet to be determined.

The datasets where DL models are known to outperform SML (i.e. Imagenet), have a relative high number of instances and low dimensionality as compared to brain imaging data. Since DL is a data-hungry technique, the question arises whether it can extract sufficient meaningful patterns out of the high dimensional data with a small amount of training data. To surpass this problem, various studies have used hand crafted input features with different levels of feature extraction along the spatial and/or temporal dimensions to reduce the input dimensionality. Although this is a practical solution to check what DL is capable of in comparison to SML, it also deprives DL of its main advantage: representation learning without feature engineering (Abrol et al., 2020). Given the endless choices in feature extraction, models and preprocessing steps, there is a large variety in DL modelling and features that have been applied to investigate psychiatric disorders. This leaves us with many questions regarding the type and input for DL applications and without any validated benchmark model.

Given the high interest in DL within the field of neuroimaging for psychiatry and the wide variety of approaches, this review aims to give an overview of studies that have applied DL to neuroimaging data for the classification of psychiatric disorders. This review will solely focus on studies related to classification of psychiatric disorders and does not include studies on other neurological disorders such as Alzheimer’s Disease (AD) as AD has been extensively reviewed recently (Jo et al., 2019, Rathore et al., 2017, Ebrahimighahnavieh et al., 2020). Moreover, the pathology of many neurological disorders, including AD, largely involves anatomical changes, whereas psychiatric disorders usually involve subtle, functional alterations that are mainly investigated through functional brain scans. In this paper we will discuss the main themes that have emerged from our review and include a quantitative comparison of the performance of deep learning and standard machine learning techniques. Finally, we will make a number of recommendations for future research.

## 2 Methods

**Fig. 3:**
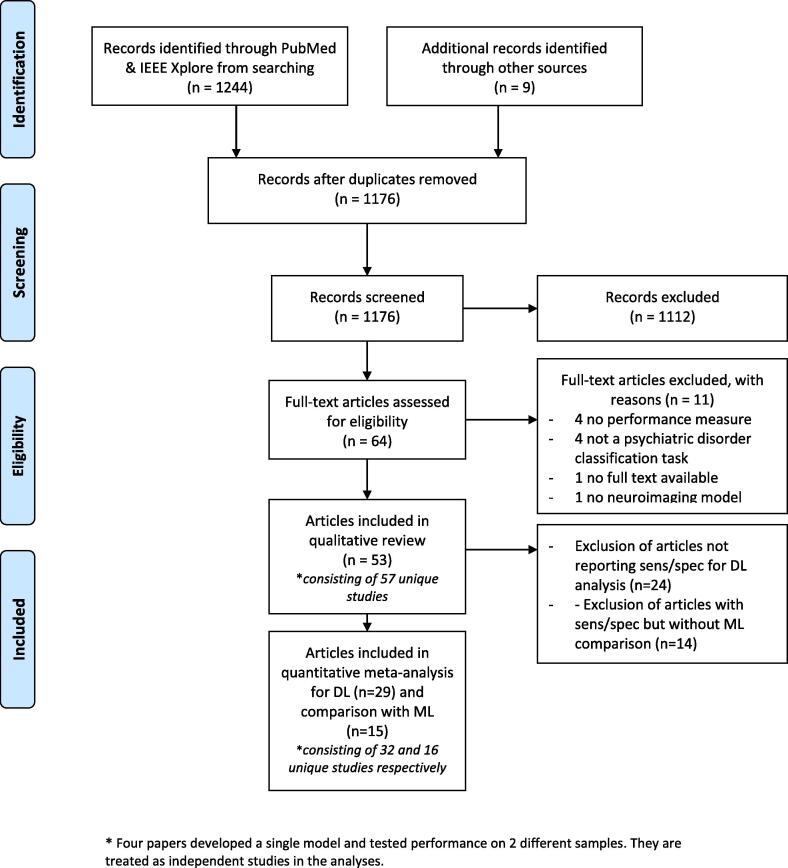
PRISMA flowchart describing the processes of literature search, study screening and selection (Moher et al., 2009).

We conducted a systematic review of published studies that used DL approaches for diagnostic classification of psychiatric disorders using neuroimaging. The search strategy is outlined in detail in the PRISMA flow diagram in Fig. 3.

### 2.1 Identification

We conducted a literature search in PUBMED and IEEE Xplore using the following search string: (“deep learning” OR “deep architecture” OR “artificial neural network” OR “convolutional neural network” OR “convolutional network” OR “CNN” OR “recurrent neural network” OR “RNN” OR “Auto-Encoder” OR “Autoencoder” OR “Deep belief network” OR “DBN” OR “Restricted Boltzmann Machine” OR “RBM” OR “Long Short Term Memory” OR “Long Short-Term Memory” OR “LSTM” OR “Gated Recurrent Units” OR “GRU”) AND (psychiatry OR psychiatric OR classification OR diagnosis OR prediction OR prognosis OR outcome) AND (neuroimaging OR MRI OR “Magnetic Resonance Imaging” OR “fMRI” OR “functional Magnetic Resonance Imaging”) which is a combination of search terms used in previous reviews on deep learning in neuroimaging (Vieira et al., 2017, Jo et al., 2019). The search was limited to articles published from the 1st of January 2013 till the 30th of September 2019.

In addition, articles in PubMed were identified that cited the previous systematic review on deep learning in neuroimaging of Vieira et al. (2017). Reference lists of identified articles were further searched to select those articles that were deemed appropriate. For the scope of this study, we excluded studies using PET or EEG, although there is some evidence that DL can be used in this type of data (Page et al., 2014). Following this approach, 1176 studies were identified.

### 2.2 Screening and inclusion

64 Articles were eligible for full-text assessment based on title and abstract screening. Articles were included if they were a peer-reviewed full-text original research article written in English using a deep learning model for classification of a psychiatric disorder using (f)MRI. Upon full manuscript reading, 11 articles were excluded due to the lack of a clear performance measure (4), not performing a classification task of a psychiatric disorder (4), lack of a full manuscript (1), and not using a deep learning model (1), yielding a total of 53 included articles. For quantitative *meta*-analysis, we included 29 articles that reported sensitivity and specificity. For comparison with SML techniques, we included 15 articles that also reported sensitivity and specificity for DL and SML.

From the 53 included papers there were 4 that developed a single model and tested classification performance for 2 different samples (different psychiatric disorders) (Sen et al., 2018, Pinaya et al., 2019, Matsubara et al., 2019, Wang et al., 2019). These papers are included twice: they are shown independently in the two corresponding tables and are analysed as independent studies, yielding a total of 57 studies for qualitative analysis, 32 for quantitative analysis for all DL studies and 16 for quantitative *meta*-analysis for DL-SML comparison.

### 2.3 Qualitative analysis

The included studies were grouped per disorder. We extracted data from all studies to compare key aspects such as sample sizes, type of features, classifier and reported accuracies. Data extraction was done by two independent researchers and discussed on consistency until agreement was reached. Next, we composed a narrative review of findings from included studies per disorder. Finally, we included visual summaries for all studies combined to discuss occurring themes in the literature.

### 2.4 Quantitative *meta*-analysis

All *meta*-analyses were conducted using the mada and metaphor package in R. As pooling sensitivities or specificities can be misleading (Gatsonis and Paliwal, 2006), we have pooled studies using diagnostic odds ratios (DOR) according to the Reitsma model and the Cochrane handbook for diagnostic tests of accuracy studies (Reitsma et al., 2005, Macaskill et al., 2010). The DOR considers both sensitivity and specificity. To visualize between study performance differences, a forest plot of the DORs with bootstrapped 95% confidence intervals is given, subdivided per disorder. In order to assess whether DL and SML models obtain different classification performances, we conducted *meta*-regression with classification method as covariate. We performed this subgroup analysis for DOR values in the metaphor package using bootstrapped confidence intervaIs. We also performed this *meta*-regression for sensitivity and false positive rates with confidence intervals provided by the mada package (Doebler and Holling, 2015). In addition, the *meta*-regression was repeated for the largest subgroups separately. Significance was set at an alpha level of 0.05.

## 3 Results

**Fig. 4:**
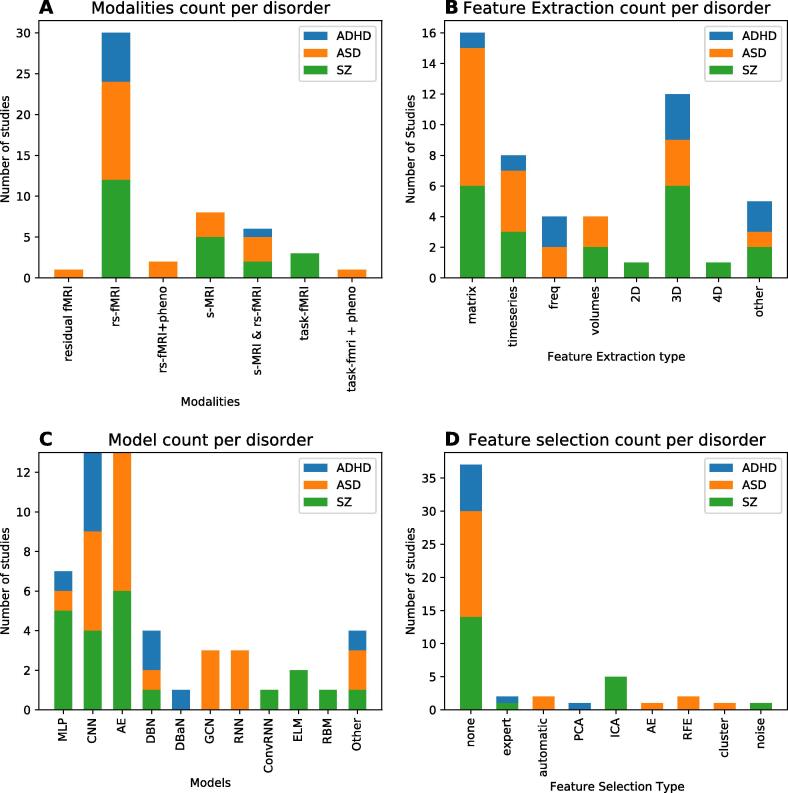
Visual summary of articles reviewed grouped by the three most investigated disorders ADHD, ASD and SZ. A) Number of articles on different modalities; B) Number of articles of different feature extraction, C) number of articles on different DL models, D) Number of articles on different feature selection procedures.

The vast majority of studies addressed the classification of autism spectrum disorder (ASD) (n = 22) or schizophrenia (SZ) (n = 22). We also retrieved 9 studies for attention-deficit/hyperactivity disorder (ADHD). Finally, we included four studies on other disorders: two studies focused on major depressive disorder (MDD), one on bipolar disorder (BD) and one on conduct disorder (CD). A summary for each study including the sample size, imaging modality, DL model, and classifier performance is presented in Table 1, Table 2, Table 3, Table 4. A visual summary of reviewed studies for ASD, SZ, and ADHD is shown in Fig. 4. As can be seen here, most studies (n = 30) used rs-fMRI as input for their DL model. The majority of rs-fMRI studies (n = 24) reduced the four-dimensional fMRI input by parcellating the brain in regions of interest (ROIs) and extracting timeseries per ROI. Most of these studies (n = 16) further reduced dimensionality by analysing correlations between ROI timeseries to create a connectivity matrix (n = 16). Fewer studies (n = 6) worked on 3D fMRI data where the time dimension had been summarized. For structural MRI (n = 11) the full 3D input data was used in slightly over half of the studies (n = 6). Finally, this summary further illustrates the large variety of models that has been deployed in this field.

**Table 1:** Overview of ASD studies included in this literature review.

Authors, year, ^ref.^	Dataset.	Sample size.	# Sites.	Input Modality.	Feature selection (y/n)	Feature engineering.	# Features.	Validation.	Best DL model.	DL ACC.	Best ML model.	ML input.	ML Acc.

Sen et al. 2018*, (Sen et al., 2018)	ABIDE.	ASD = 573, TD = 538.	17.	s-MRI & rs-fMRI.	no.	Unsup. features (structural + spatio-temporal)	45 IC for fMRI + ? sMRI.	5 cv on training / 1 test.	multimodal feature learning + linear SVM.	64.3.	x.	x.	x.
Pinaya et al. 2019**, (Pinaya et al., 2019)	HCP, ABIDE.	pretraining HC = 1113; ASD = 83, HC = 105.	17.	s-MRI.	no.	Freesurfer cortical thickness and anatomical volumes.	x (Freesurfer 104 regions)	10 strat cv.	AE.	63.9^a^	SVM (lin)	Freesurfer cortical thickness and anatomical volumes.	56.9 ^a^
Aghdam et al. 2018, (Aghdam et al., 2018)	ABIDE I + II.	ASD = 116, TD = 69.	7.	s-MRI & rs-fMRI.	no.	mean of AAL tc + GM/WM AAL parcellation.	232 or 348.	10 cv.	DBN.	65.6.	x.	x.	x.
Xing et al. 2018, (Xing et al., 2018)	ABIDE I.	ASD = 527, TD = 569.	17.	rs-fMRI.	no.	AAL (90) FC matrix.	4005.	10x strat 5 cv.	CNN_EW.	66.9.	SVM.	AAL (90) FC matrix.	63.6.
Ktena et al., 2018,(Ktena et al., 2018)	ABIDE.	ASD = 403, TD = 468.	20.	rs-fMRI.	no.	anatomical spatial graphs with labels of HO FC matrix.	x.	5 cv.	GCN.	~67.	PCA/Euclidean.	anatomical spatial graphs with labels of HO FC matrix.	~54.
Li et al. 2018, (Li et al., 2018)	ABIDE-UM.	ASD = 48, TD = 65 (+411 training)	17*	rs-fMRI.	no.	AAL (90) FC matrix.	4005.	strat 5 cv.	SSAE-DNN.	67.2.	SVM.	AAL (90) FC matrix.	60.5.
Kam et al. 2017, (Kam et al., 2017)	ABIDE I UM NYU.	ASD = 119, TD = 144.	2.	rs-fMRI.	yes, hierarchical cluster^!^	AAL FC matrix.	x.	train/test.	DRBM.	67.4.	SVM (graph theory)	AAL FC matrix.	65.9.
Dvornek et al., 2017, (Dvornek et al., 2017)	ABIDE I.	ASD = 529, TD = 571.	17.	rs-fMRI.	no.	CC200 tc.	90*200.	10 strat cv.	LSTM.	68.5.	x.	x.	x.
Dvornek et al. 2018, (Dvornek et al., 2018)	1 site.	ASD = 21, TD = 19.	1.	task-fMRI + pheno.	no.	timeseries AAL(90) atlas.	156*90 timeseries.	10x 10 cv.	LSTM.	69.8.	x.	x.	x.
Heinsfeld et al.2018, (Heinsfeld et al., 2018)	ABIDE I.	ASD = 505, TD = 530.	17.	rs-fMRI.	no.	CC200 FC matrix.	19,900.	10 cv and leave-site out.	AE-MLP.	70.	SVM.	CC200 FC matrix.	65.
Dvornek et al. 2018, (Dvornek et al., 2018)	ABIDE I.	ASD = 529, TD = 571.	17.	rs-fMRI + pheno.	no.	CC200 tc.	90*200 tc + 90*5 phenotypic data.	10 site-strat cv.	Pheno_LSTM.	70.1.	x.	x.	x.
Parisot 2018, (Parisot et al., 2018)	ABIDE I.	ASD = 403, TD = 468.	20.	rs-fMRI + pheno.	yes, RFE.	HO (1 1 0) FC matrix + pheno(sex, site)	2000.	10 strat cv.	GCN.	70.4.	ridge.	HO (1 1 0) FC matrix + pheno(sex, site)	65.3.
Aghdam et al. 2019, (Aghdam et al., 2019)	ABIDE I + II.	ASD = 210, TD = 249.	20.	rs-fMRI.	no.	Max freq. voxel level.	2D images of (~70*95)	10 cv.	combined mixed expert CNN.	70.5.	x.	x.	x.
Anirudh & Thiagarajan 2019, (Anirudh and Thiagarajan, 2017)	ABIDE I.	ASD = 403, TD = 468.	20.	rs-fMRI.	no.	HO (1 1 0) FC matrix + pheno(sex, site)	x.	10 cv.	ensemble G-CNN.	70.9.	SVM(lin)*	FC matrix.	66.8.
Khosla et al. 2018, (Khosla et al., 2019)	ABIDE I.	ASD=379, TD=395.	17.	rs-fMRI.	no.	multi-channel 3D voxel connectivity maps.	x.	10 cv (and ABIDE I/II split)	ensemble 3D CNN.	73.5.	SVM(RBF)	FC matrix.	71.
Li et al. 2018, (Li et al., 2018)	NDAR.	ASD=61, TD=215.	unclear.	s-MRI.	yes, discriminative landmarks (automatic)^!!^	50 3D volumes + pheno info (sex, WB volume)	24x24x24x50	10 cv	CNN	76.24	x	x	x
Mellema et al. 2019, (Mellema et al., 2019)	IMPAC	ASD=418, TD=497	unclear	s-MRI & rs-fMRI	no	FC matrix + ROI volumes	x	Strat. 3cv	MLP	80.4 ^a^	Logistic Ridge Regression	FC matrix + ROI volumes	77.34^a^
Guo et al. 2017, (Guo, 2017)	ABIDE UM	ASD=55, TD=55	1	rs-fMRI	yes, based on SAE	AAL FC matrix - feature selection based on multiple SAE	6670	nested 5 cv	SAE-DNN	86.4	Elastic net	AAL FC matrix	79,5
Dekhil et al. 2018, (Dekhil, et al., 2018)	NDAR	ASD=123. TD=160	2	rs-fMRI	no	PSD of tc of 34 gICA ROIs	34*83	2,4,**10 cv** and LOO-CV with 100 permutations	SAE_SVM	91	PCA_SVM	PSD of tc of 34 gICA ROIs	84
Li et al. 2018, (Li et al., 2018)	1 site	ASD=82, TD=48	1	residual f-MRI	no	2 channel (mean and std) 3D volumes	2*32x32x32=65536	Strat. 4 cv	2-channel 3DCNN	89^b^	RF	flattened vector of 2 channel 3D volumes (65536 dimensions) + PCA	82^b^
Ismail et al. 2017, (Ismail, 2017)	KKI	ASD=21, TD=21	1	s-MRI	yes, ROIS (automatic)	CDF of 64 shape features	64*4000	train/test	SAE	92.8	x	x	x
Wang et al. 2019, (Wang et al., 2019)	ABIDE I	ASD=501, TD=553	17	rs-fMRI	yes, top 1000 of RFE^!^	AAL (1 1 6) FC matrix	6670	average of 5,10,20,30 cv	SVM-RFE + SSAE	93.6	SVM-RFE + softmax classifier	AAL (1 1 6) FC matrix	67.3

* General model for ASD and ADHD, ** General model for ASD and SZ

^a^ AUC ROC, ^b^ F score, ^c^ Balanced accuracy

^!^ not clear if feature selection is done only on training set, ^!!^ Feature selection done before train/test split

ASD = Autism Spectrum Disorder, TD = typically developing, rs = resting state, fMRI = functional Magnetic Resonance Imaging, s-MRI = Magnetic Resonance Imaging, ABIDE = Autism Brain Imaging Data Exchange, NDAR = National Database for **Autism** Research, IMPAC = Maging-PsychiAtry Challenge, UM = University of Michigan, KKI = Kennedy Krieger Institute , PSD = Power Spectral Densities, Tc = timecourse, gICA = group Independent Component Analysis, NMI = Normalized Mutual Information, CDF = cumulative distribution function, WB = whole brain, PCA = principle component analysis, SVM = support vector machine, AAL = automatic anatomic labelling, CC200, craddock 200, HO = Harvard Oxford, ROI = Region of interest, CNN = convolutional neural network, EW = element-wise filter, GCN = grapch convolutional network, AE = Auto Encoder, SAE = Stacked Auto encoder, SSAE = stacked sparse auto encoder, RF = random forest, MLP = multilayer perceptron, LSTM = long short-term memory, DBN = Deep belief network, DRBM = Deep restricted Boltzmann machine, FC = functional connectivity, 10 cv = 10 fold cross validation, LOOCV = leave one out cross validation, strat cv = stratified cross validation

**Table 2:** Overview of SZ studies included in this literature review.

Authors, year, ^ref^	Dataset	Sample size	# Sites	Input Modality	Feature selection (y/n)	Feature engineering	# Features	Validation	Best DL model	DL Acc	Best ML model	ML input	ML Acc
Dakka et al. 2017, (Dakka et al., 2017)	1 site	SZ = 46, HC = 49	1	task-fMRI	no	full 4D image	x	10 cv	LSTM	66.4	SVM (rbf)	4D reduced to 1D vector	62.1
Pinaya et al. 2019***, (Pinaya et al., 2019)	HCP, NUSDAST	pretraining HC = 1113 ; SZ = 35,HC = 40	1	s-MRI	no	Freesurfer cortical thickness and anatomical volumes	x (Freesurfer 104 regions)	10 strat cv	AE	70.7^a^	SVM (lin)	Normalized Freesurfer cortical thickness and anatomical volumes	63.7^a^
Matsubara et al. 2019*, (Matsubara et al., 2019)	openfMRI	SZ = 48, HC = 117	1	rs-fMRI	no	AAL timeseries	116*152	10 cv	DGM (CVAE)	71.3^c^	PCC_SCCA_SLR	AAL FC matrix	66.4^c^
Vyskovsky et al. 2019, (Vyskovsky et al., 2019)	1 site	SZ = 52, HC = 52	1	s-MRI morphometry	yes, discriminative features^!^	VBM and DBM Grey Matter Images	100–10.000	10x LOOCV	ensemble MLP for VBM and DBM	73.1	SVM on VBM and DBM	VBM, DBM	73.5
Pinaya et al. 2016, (Pinaya et al., 2016)	1 site	SZ = 143, HC = 83	1	s-MRI		Freesurfer cortical thickness and anatomical volumes	x	3 cv	DBN-DNN	73.6^c^	SVM	Freesurfer cortical thickness and anatomical volumes	68.1^c^
Ulloa et al., 2015, (Ulloa et al., 2015)	JHU, MPRC, IOP, WPIC												
	SZ = 198, HC = 191	4	s-MRI	no	generating sMRI images with RV generator	55,527	10 cv	sMRI generator + MLP	75 ^a^	Logistic Regression	sMRI images	70 ^a^	
Han et al. 2017, (Han et al., 2017)	1 site	SZ (Sheffield and Barch, 2016) = 39, HC = 31	1	rs-fMRI	no	AAL (90) FC matrix	4005	10 cv	MLP	79.3	x	x	x
Li et al. 2019, (Li, 2019)	1 site	SZ = 80, HC = 103	1	task fMRI and SNP	no	SNP loci from blood + AAL ROI	116	Train/test	2 SAE + DCCA + SVM	80.5	x	x	x
Lei et al. 2019, (Lei et al., 2020)	5 sites	SZ = 295, HC 452	5	rs-fMRI	no	FC matrix 90 ROIS	4005	strat 5 cv	2D CNN	81.0^c^	SVM	FC matrix 90 ROIS	81.7^c^
Wang et al. 2019**, (Wang et al., 2019)	1 site	SZ = 28, HC = 28	1	rs- fMRI	no	based on a single 3D EPI image	61*73*61	5 cv	3D CNN	82.2	x	x	x
Yang et al. 2019, (Yang et al., 2019)	COBRE, UCLA, WUSTLE	SZ = 102, HC = 120	3	rs-fMRI	no	3 ensemble inputs: sparse dictionary learning, multiple kernel mapping, AAL FC matrix	80*20; 100*50; 116*116	10 cv	ensemble capsule network	82.8	weighted ensemble SVM	3 ensemble inputs: sparse dictionary learning, multiple kernel mapping, AAL FC matrix	74.2
Yan et al. 2019, (Yan et al., 2019)	7 sites	SZ = 558, HC = 542	7	rs-fMRI	yes, group ICA noise^!!^	group ICA tc	8500 (170 TR * 50 IC)	10 cv and LSO	Conv + RNN	83.2	SVM	group ICA FC matrix (50*50)	79.4

**Authors, year, ^ref^**	**Dataset**	**Sample size**	**# Sites**	**Input Modality**	**Feature selection (y/n)**	**Feature engineering**	**# Features**	**Validation**	**Best DL model**	**DL Acc**	**Best ML model**	**ML input**	**ML Acc**
Oh et al. 2019, (Oh et al., 2019)	1 site	SSD = 103, HC = 41	1	task-fMRI	no	3D GLM activation map	x	10 cv	3D CAE-CNN	84.4	SVM + PCA	3 ways: full WB, beta AAL, 40 PCA features	70.7
Yan et al. 2017, (Yan, 2017)	7 sites	SZ = 558, HC = 542	7	rs-fMRI	yes, group ICA noise^!!^	group ICA FC matrix (50*50)	1225	10 cv and LSO	DNN + LRP	84.8	SVMRFE	group ICA FC matrix (50*50)	77.1
Zeng et al. 2018, (Zeng et al., 2018)	COBRE, UCLA, WUSTL, XJING1_2, AMU, Xiangya	SZ = 357, HC= 377	7	6 rs-fMRI, 1 task fMRI	no	FC of diff atlases(ROI: 176, 160, 116)		10 cv + leave site out validation	DANS with 3 atlas features fusion at label level	85.0	RFE-LDA	selected features from correlation matrices 3 atlases label level fusion;	80.9
Kim et al. 2016, (Kim et al., 2016)	COBRE	SZ= 50, HC=50	1	rs-fMRI	no	group ICA FC matrix (116*116)	6670	10 x nested 5 cv	2 SAE + DNN	86.5	SVM (lin)	FC matrix GICA	76.9
Plis et al. 2014, (Plis, 2014)	JHU, MPRC, IOP, WPIC	SZ = 198, HC=191	4	s-MRI	no	RBM feature learning	60,645 voxel GM images	10 cv	RBM of 3 layers + Logistic regression for classification	91^b^	x	x	x
Chyzhyk 2015, (Chyzhyk et al., 2015)	COBRE	SZ=72, HC=74	1	rs-fMRI	Yes, evolutionary selection algorithm	VHMC map	86,559	10 cv	Ensemble of ELM	91.2	RF on ReHo	ReHO selected C map	80.9
Patel 2016, (Patel et al., 2016)	COBRE	SZ=72, HC=74	1	rs-fMRI	yes, filter out inactive or noisy GM voxels	AAL (116) timeseries		10 cv	SAE_SVM	92	x	x	x
Srinivasagopalan 2019, (Srinivasagopalan et al., 2019)	Kaggle dataset	SZ = 69, HC=75	1	s-MRI & rs-fMRI	yes, ICA noise selection	FC maps ICA brain maps derived from GM concentration	411	Train/test	MLP	94.4	RF	55 selected features with RFE and RF	83.3
Qureshi et al. 2019, (Qureshi et al., 2019)	COBRE	SZ = 72, HC=72	1	rs-fMRI	yes, group ICA noise^!^	3D-ICA	15	10 cv	3DCNN	98.0	x	x	x
Qureshi et al. 2017, (Qureshi et al., 2017)	COBRE	SZ = 72, HC=72	1	s-MRI & rs-fMRI	yes, group ICA noise^!^	structural ROI, global functional connectivity, group ICA, kernel PCA with spatial ICA maps	748	nested 10 by 10 cv	ELM	99.3	SVM-L	structural ROI, global functional connectivity, group ICA, kernel PCA with spatial ICA maps	77.8

* General model SZ and BD, ** General model SZ and ADHD, *** General model SZ and ASD

SZ (Sheffield and Barch, 2016) early onset Schizophrenia

^a^AUC ROC, ^b^ F score, ^c^ Balanced accuracy

^!^ not clear if feature selection is done only on training set,^!!^ Feature selection done before train/test split

SZ = Schizophrenia, HC = healthy controls, rs = resting state, fMRI = functional Magnetic Resonance Imaging, s-MRI = Magnetic Resonance Imaging, Tc = timecourse, gICA = group Independent Component Analysis, GM = grey Matter,WB = whole brain, VBM = voxel based morphometry, DBM = dephormation based morphometry, SNP = single nucleotide polymorphisms, PCA = principle component analysis, SVM = support vector machine, AAL = automatic anatomic labelling, CC200, craddock 200, HO = Harvard Oxford, ROI = Region of interest, VHMC = voxel-mirrored homotopic connectivity, CNN = convolutional neural network, EW = element-wise filter, GCN = grapch convolutional network, GLM = General linear model, AE = Auto Encoder, DGM = deep generative model, CVAE = conditional variational auto encoder, SAE = Stacked Auto encoder, SSAE = stacked sparse auto encoder, CAE = convolutional auto encoder, ReHo = Regional Homogeneity, RF = random forest, MLP = multilayer perceptron, LDA = linear discriminant analysis, LSTM = long short-term memory, LRP = Layer wise relevance propagation, DBN = Deep belief network, RNN = recurrent neural network, RBM = Restricted Boltzmann Machine, DANS = Discriminant Autoencoder Network with Sparsity Constraint, ELM = Extreme Learning Machine, FC = functional connectivity, 10 cv = 10 fold cross validation, LOOCV = leave one out cross validation, strat cv = stratified cross validation, LSO = leave site out, COBRE = Center for Biomedical Research Excellence, JHU = Johns Hopkins University, MPRC = the Maryland Psychiatric Research Center, IOP = the Institute of Psychiatry, WPIC = Western Psychiatric Institute and Clinic at the University of Pittsburgh, UCLA = university of california Los Angeles, WUSTL = Washingthon university in st. Louis, AMU = Anhui Medical University, HCP = Humman Connectome Project, NUSDAST = Northwestern University Schizophrenia Data and Software Tool

**Table 3:** Overview of ADHD studies included in this literature review.

Authors, year, ^ref^	Dataset	Sample size	# Sites	Input Modality	Feature selection (y/n)	Feature engineering	# Features	Task	Validation	Best DL model	DL ACC	Best ML model	ML input	ML ACC
Kuang et al. 2014, (Kuang et al., 2014)	ADHD-200-NYU	HC = 107, ADHD-C = 99, ADHD-I = 44, ADHD-H = 13	1	rs-fMRI	yes (expert)	ROI (PFC) max freq	x	HC vs. ADHD-C vs ADHD-I vs ADHD-H	Train/test	DBN	37.4	x	x	x
Kuang and He, 2014, (Kuang and He, 2014)	ADHD-200	HC = 160, ADHD-C = 125, ADHD-I = 50, ADHD-H = 14	3	rs-fMRI	no	WB freq PCA	257*9177	HC vs. ADHD-C vs ADHD-I vs ADHD-H	Train/test	DBN	44.6	x	x	x
Hao et al., 2015, (Hao et al., 2015)	ADHD-200_NYU	HC = 110, ADHD-C = 95, ADHD-I = 2, ADHD-H = 50	1	rs-fMRI	no	selected ROI network of 14 ROIS	x	HC vs. ADHD-C vs ADHD-I vs ADHD-H	100 cv	DBaN	64.7	x	x	x
Sen et al., 2018*, (Sen et al., 2018)	ADHD-200	ADHD = 356, HC = 373	8	s-MRI & rs-fMRI	no	Unsupervised features (structural + spatio-temporal)	45 IC for fMRI + sMRI	ADHD vs TPC	Train/test	Multimodal feature learning + linear SVM	67.3	x	x	x
Wang & Kamata, 2019, (Wang and Kamata, 2019)	ADHD-200	ADHD = 362, HC = 585	7	s-MRI	no	3D fractal dimension complexity map (FDCM)	96*120*100	ADHD vs TPC	Train/test	3D CNN	69.0	x	x	x
Zou et al. 2017, (Zou et al., 2017)	ADHD-200	ADHD = 197, HC = 362	8	rs-fMRI	no	ReHo, fALFF, VMHC	3 * 47 * 60 * 46 + 3 * 90*117 *100	ADHD vs TPC	10 cv and leave-site out	3D CNN	69.2	x	x	x
Riaz et al., 2018, (Riaz, et al., 2018)	ADHD-200	HC = 95, ADHD-C = 127*	1	rs-fMRI	no	90 AAL timeseries	900*T	HC vs ADHD	Train/test	CNN	73.1	SVM	FC matrix with feature selection of elastic net	56.1
Wang et al., 2019**, (Wang et al., 2019)	ADHD-200	ADHD = 146, HC = 441	8	s-MRI	no	full 3D image	121*145*121	ADHD vs TPC	5 cv	3D CNN	76.6	x	x	x
Desphande et al., 2015, (Deshpande et al., 2015)	ADHD-200	HC = 744, ADHD-C = 260, ADHD-I = 173	7	rs-fMRI	yes, (PCA)	200 PCA connectivity features	20	HC vs ADHD-C	LOOCV	Fc cascade NN with 2 training stages	~90	SVM	significant features of PCA + conn weights	~80

* General model for ADHD and ASD, ** General model for ADHD and SZ

^a^AUC ROC, ^b^ F score, ^c^ Balanced accuracy

^!^ not clear if feature selection is done only on training set, ^!!^ Feature selection done before train/test split

ADHD = Attention Deficit hyperactivity disorder, -I, Inattentive, -H hyperactive, -C combined, HC = healthy control, rs = resting state, fMRI = functional Magnetic Resonance Imaging, s-MRI = structural Magnetic Resonance Imaging, AAL = automatic anatomic labelling, ROI = Region of interest, CNN = convolutional neural network, DBN = Deep belief network, DBaN = deep baysesian network, FC = functional connectivity, SVM = support vector machine, 10 cv = 10 fold cross validation, LOOCV = leave one out cross validation, PCA = principle component analysis, ReHO = regional homogeneity, VHMC = voxel-mirrored homotopic connectivity, fALFF = Fractional amplitude of low-frequency fluctuations, NN = neural network.

**Table 4:** Overview of BD, CD, MDD studies included in this literature review.

Authors, year, ^ref^	Disorder	Dataset	Sample size	# Sites	Input Modality	Feature selection (y/n)	Feature engineering	# Features	Task	Validation	Best DL model	DL Acc	Best ML model	ML input	ML Acc
Matsubara et al., 2019*, (Matsubara et al., 2019)	Bipolar Disorder (BD)	openfMRI	BD = 46, HC = 117	1	rs-fMRI	no	AAL timeseries	116*152	BD vs HC	10 cv	DGM (CVAE)	64.0^c^	PCC_Kendall_LLE_Cmeans	AAL FC matrix	62.2^c^
Zhang et al. 2019, (Zhang et al., 2020)	Conduct disorder (CD)	1 site	CD = 60, HC = 60	1	s-MRI	no	full 3D image with augmentation	121*145*121	CD VS HC	5 cv	3D CNN	85	SVM(lin)	VBM	77
Pominova et al., 2018, (Pominova, 2018)	Major Depressive Disorder (MDD)	1 site	MDD = 25, HC = 25	1	rs-fMRI	yes, cleaned data (unclear)	full 4D image	52*62*52*133	MDD vs HC	5 cv	3DConvLSTM	73	x	x	x
Miholca & Onicas, 2017, (Miholca and Onicaş, 2017)	Major Depressive Disorder (MDD)	openfMRI	MDD = 19, HC = 20	1	task-fMRI	yes, task related ROI^II^	task-related param. of selected ROIs	x	MDD vs HC	LOOCV	MLP	92.3	RAR based classifier	task-related param. of selected ROIs	94.8

* General model for BD and SZ

^a^AUC ROC, ^b^ F score, ^c^ Balanced accuracy

^!^not clear if feature selection is done only on training set, ^!!^ Feature selection done before train/test split

BD = Bipolar Disorder, CD = Conduct Disorder, MDD = Major Depressive Disorder, HC = healthy control, rs = resting state, fMRI = functional Magnetic Resonance Imaging, s-MRI = Magnetic Resonance Imaging, AAL = automatic anatomic labelling, ROI = Region of interest, DGM = Deep neural generative model, CVAE = conditional variational auto encoder, CNN = convolutional neural network, ConvLSTM = convolutional Long Short-Term Memory, MLP = multilayer perceptron, FC = functional connectivity, SVM = support vector machine, RAR = Relational association rules, VBM = Voxel based morphometry, LLE = locally linear embedding, 10 cv = 10 fold cross validation, LOOCV = leave one out cross validation

### 3.1 Autism spectrum disorder (ASD)

Twenty-two studies have applied DL for classification of ASD with accuracies ranging from 50 to 94. As shown in Table 1, eighteen studies have used data from the Autism Brain Imaging Data Exchange (ABIDE), a data-sharing initiative involving >20 different scanning sites. The ABIDE features over 2000 structural and functional MRI scans of autistic and typically developing children and came out in two releases: ABIDE-I and II. Even though these studies have used the same dataset, there is a large difference in subsets used, with sample sizes ranging from 110 to 1054. As shown in Fig. 4, three studies have used structural MRI (s-MRI) as input. Li et al. (2018) applied 3D CNNs on s-MRIs of the national database for autism research (NDAR) (n = 276) and achieved 76.2% accuracy. Pinaya et al. (2019) used a deep autoencoder to pre-train s-MRI data of the human connectome project (HCP) to detect alterations in the ABIDE and achieved an AUC-ROC of 0.64 (n = 188). The study with the highest reported accuracy using s-MRI of Ismail et al., 2017) used a stacked auto encoder (SAE) on cumulative distribution function (CDF) of shape features and reached up to 92.8% accuracy. However, this is also the study with the smallest study size (n = 42) and they did not report cross-validated results.

Of the studies using fMRI, the vast majority used resting state (rs-) fMRI. Instead, Li et al. (2018) used residual fMRI: task f-MRI controlled for the task-related signal. They used a 3D convolutional model on 3D brain volumes where the time dimension is summarized in mean and standard deviation of voxel’s timecourses per time window. With this approach they obtained 89% accuracy, the highest accuracy for studies without any feature selection. As can be seen in Table 1, when accuracy performances are getting higher (lower in the Table), feature selection is done more often. One needs to be careful with concluding that feature selection is beneficial for performance, as it seems that several studies have done feature selection on the whole sample instead of properly selecting features only on the training set. Wang et al. (2019) reported a very high accuracy of 93.6 on the full ABIDE I dataset, using a stacked sparse autoencoder on selected features of a functional connectivity (FC) matrix. They applied SVM-RFE for the selection of 1000 features. However, this appears to be done on the entire dataset without keeping the test set separately. This increases the risk of overfitting, complicates model interpretation, and may produce optimistic results (Mwangi et al., 2014, Varoquaux et al., 2017, Scheinost et al., 2019).

Besides Wang et al. (2019), seven other ASD studies have applied a DL model on FC matrices, making it the most common input feature used in DL classifications for ASD. Mainly the Automated Anatomic Labeling (AAL), Craddock or Harvard-Oxford (HO) atlas are used, probably because the ABIDE provides extracted timecourses for these atlas parcellations. Interestingly, most studies on the ABIDE-I achieve similar accuracies, ranging from 65 to 71%, with intrinsically different methods. On a single site of the ABIDE dataset consisting of 110 samples, Guo et al. (2017)) achieved an accuracy of 86,4 using an autoencoder to pretrain an MLP. Li et al. (2018) also pretrained an MLP with stacked autoencoders, but obtained an accuracy of 67.2% when training on multiple sites and testing on one. With a similar approach of pretraining an MLP, Heinsfeld et al. (2018) obtained an accuracy of 70% when testing on the full ABIDE-I release, consisting of 1035 samples.

Whereas these studies reshape the connectivity matrix into a vector, Xing et al. (2018) retained spatial information of the network topology by applying convolutional networks (CNN) to the full FC matrix. Their results on the full ABIDE-I results are similar to Heinsfeld et al. (2018), reaching 66.88 with their best CNN model. Graph convolutional approaches are explored by Parisot et al., 2018, Anirudh and Thiagarajan, 2017, obtaining 70.4% and 70.9% accuracy respectively. Using a discriminative restricted Boltzmann machine (DRBM), Kam et al. (2017) reached an accuracy of 67.4% on two sites of the ABIDE dataset.

Instead of focusing on FC matrices, four studies have incorporated the time dimension and worked on timeseries as input data (Dvornek et al., 2018b, Dvornek et al., 2018c, Dvornek et al., 2017, Dekhil, et al., 2018). In three different studies by (Dvornek et al., 2017, Dvornek et al., 2018b, Dvornek et al., 2018c), they have experimented with the optimal input for Long Short-Term Memory (LSTM) models. The highest performance on timeseries input is reported by a study from Dekhil et al. (2018). They transformed timeseries into power spectral densities (PSD) for 34 group independent component analysis (ICA) spatial maps and used sparse auto encoders (SAE) to reduce the input dimensionality so it could be fed into an SVM. They obtained a high accuracy of 88%, but on a relatively homogeneous dataset with 2 different scanning sites (as compared to > 20 in ABIDE).

There are three studies that have incorporated both structural and functional MRI as input to the DL model (Sen et al., 2018, Aghdam et al., 2019, Mellema et al., 2019) reported the highest accuracy of 80.4% on a large dataset (n = 915) by inputting FC and ROI volume values into an MLP. A major part of their success seems to be due to their multimodal input, as even a simplistic logistic regression obtained an accuracy of 77.3. Finally, there are three studies that have worked on 3D input data (Khosla et al., 2019, Li X. et al., 2018, Li G. et al., 2018). Khosla et al. (2019) used the largest, most heterogeneous dataset (n = 774, sites = 17) and achieved 73.5% by using an ensemble of 3D Convolutional Neural Networks.

Overall, a wide variety of input, models and subsets of the data has been used, making it difficult to make direct comparisons between studies.

### 3.2 Schizophrenia (SZ)

Similar to the other disorders, the first papers on deep learning for schizophrenia classification appeared in 2016 and in the last 3 years many papers have followed. We included twenty-two studies for SZ classification with an accuracy range of 66–99 that are shown in Table 2. In contrast to ASD, there is a large variety in datasets used despite different data sharing efforts such as the MCIC and COBRE. Most sample sizes are relatively small as compared to the ABIDE or ADHD-200. The largest studies of (Yan, 2017, Yan et al., 2019) with a cohort of 1100 subjects report accuracies over 80%, which is relatively high as compared to the classification performances on the full ABIDE dataset. Yet, the SZ sample may be more homogeneous as it only consists of seven different scanning sites. (Yan, 2017, Yan et al., 2019) have reported a model on FC matrices of group independent component analysis (ICA) spatial maps as well as its timeseries. Their first model on the FC matrices using an MLP outperformed a Convolutional Recurrent Network on timeseries, but the difference is small: 84.8% vs 83.2%. It seems that both studies have done group ICA to select spatial brain components on the whole sample before splitting the data into training and test sets. Although the influence of including test data in group ICA for spatial maps may be minimal, it is preferred to keep the training and test data completely separate, to prevent model ‘peeking’ into test data and making it more susceptible to overfitting (Scheinost et al., 2019).

We suspect that model ‘peeking’ has biased the results of two studies of (Qureshi et al., 2017, Qureshi et al., 2019b) which report the highest classification accuracies for SZ. They performed ICA on brain-wise correlation coefficients to select functional networks. The authors state that after ICA, specific components are ‘discarded as noise and/or artifacts upon visual inspection’ and it is not explicitly mentioned whether this is done on the training set only. On a multimodal input of structural MRI features including cortical thickness, surface area, volume, white matter volume and intensity measures from cortical parcellation and fMRI features consisting of these ICA selected global connectivity maps, they report a classification accuracy of 99.3% on the COBRE dataset (Qureshi et al., 2017). In a second study the performance dropped minimally to 98.1% (Qureshi et al., 2019) when applying 3D convolution neural networks on 3D volumetric images of the same group ICA selected connectivity maps.

There are two other studies applying a convolutional network, both reporting accuracies over 80% (Lei et al., 2020, Oh et al., 2019) with different approaches. (Lei et al., 2020) experimented with different inputs but obtained the highest results with a 2D CNN on FC matrices. (Oh et al., 2019) Oh et al. (2019) used a 3D convolution autoencoder on 3D activation maps based on contrast images (activation vs. control) derived from task-fMRI.

Besides Qureshi et al. (2017), one other study of Srinivasagopalan et al. (2019) used a multimodal input from structural and resting-state functional MRI made available by a classification competition (Silva, 2014). Features included FC values and source-based Morphometry (SBM) loadings; the latter corresponds to the weights of brain maps obtained from ICA on gray-matter concentration maps. They achieved an accuracy of 94.4% with a normal MLP of 3 layers on all 411 features from FC and SBM which outperformed traditional machine learning techniques as logistic regression, SVM and random forest (Srinivasagopalan et al., 2019).

Seven studies have used the COBRE dataset, of which the highest accuracies reported are from (Qureshi et al., 2017, Qureshi et al., 2019b), followed by Patel et al. (2016) with an accuracy of 92%. They trained an SAE on each ROI timeseries to obtain an encoded vector that could be fed into an SVM. Chyzhyk et al. (2015) obtained a similarly high accuracy of 91% with a very different approach; they used an evolutionary algorithm for feature selection of 3D voxel-mirrored homotopic connectivity (VHMC) maps. This input was fed into an ensemble of extreme learning machines (ELM) for classification. Yang et al. (2019) also used an ensemble of networks to classify an input of multiple image features (including functional connectivity, nonlinear multiple kernel learning and sparse dictionary learning) and obtained 82.8% accuracy on 3 datasets including COBRE. Kim et al. (2016) used a deep learning technique to select features that could be passed on to a standard machine learning model: they used a stacked auto encoder on timeseries from the AAL atlas to encode a latent feature vector that was fed into an SVM to obtain an accuracy of 86.5%. Similarly, Zeng et al. (2018) selected discriminative features from correlation matrices using an autoencoder that were parsed into an SVM for classification. On a sample from 7 datasets, including COBRE, they achieved 85% accuracy with their best model.

Remarkably, one study focusing only on structural MRI by Plis et al. (2014) also obtained a relatively high F score of 0.91 using restricted Boltzmann machine (RBM) on 2D gray matter voxel images on a larger dataset (n = 389).

One study applied transfer learning; the normative model of Pinaya et al. (2019) (also mentioned in the ASD section) trained on data from the human connectome project (HCP), was not only tested on ASD data, but also detected neuroanatomical deviations in SZ patients, reaching an accuracy of 70.7% for SZ.

### 3.3 ADHD

We included nine studies on ADHD classification. As shown in Table 3, they all have used the ADHD-200 dataset. Nevertheless, sample size varies and ranges from 349 to 1167 subjects. Three studies have performed classification of the ADHD subtypes (inattentive, hyperactive or both) with accuracies ranging from 27 to 65 (chance level of 25% for classification of 4 different groups). The highest performance for subtype classification is reported by Hao et al. (2015) that achieved 64.7% on a constructed Bayesian network on the max frequencies ROIS from rs-fMRI data. For bivariate classification of ADHD the highest accuracy is reported by Deshpande et al. (2015). They used a fully connected cascade neural network on 200 spatial PCA connectivity features and obtained around 90% accuracy.

Using a convolutional neural network on structural MRI, (Wang et al., 2019) applied 3D convolutions and obtained an accuracy of 77.6%. They also tested their model on SZ data and obtained an accuracy of 82.2% for SZ. One other study by Sen et al. (2018) developed one model that was tested on two psychiatric disorders; they developed an autoencoder to learn features from structural MRI and ICA to learn spatial features from fMRI. These combined learned features were classified by an SVM classifier and tested on ADHD and ASD to obtain 68% and 63% respectively.

Three other studies deployed convolutions for classification (Wang and Kamata, 2019, Zou et al., 2017), all with different inputs: AAL timeseries (Riaz, et al., 2018), a combination of ReHo, fALFF and VHMC (Zou et al., 2017) or 3D structural maps 68. There does not appear to be a large difference between using rs-fMRI or structural MRI in these studies, but they are difficult to compare as they have used different subsets of the ADHD-200 and applied different validation procedures.

Remarkably, four out of nine studies do not perform cross-validation but train their model once on training data and then report the performance on test data (Wang and Kamata, 2019, Riaz, et al., 2018, Kuang et al., 2014, Kuang and He, 2014). This might be since the ADHD-200 dataset started off as a competition and provides this train/test split.

### 3.4 Other disorders

We included four studies that investigated classification of other disorders, which are summarized in Table 4. These four studies have relatively small sample sizes, ranging from 49 to 163. One study of Matsubara et al. (2019) developed a single model for classification of fMRI data and tested this for both schizophrenia and bipolar disorder (BD). They used the AAL timeseries and obtained a balanced accuracy of 64% for BD (and 71.3% for SZ). Zhang et al. (2020) applied 3D convolutions on structural MRI to classify conduct disorder (CD) with an accuracy of 85%. Two studies classified major depressive disorder (MDD) (Pominova, 2018, Miholca and Onicaş, 2017). Miholca and Onicas (2017) obtained an accuracy of 92% using an MLP on task fMRI, but they selected features on the whole dataset, including test data. Pominova et al. (2018) (Pominova, 2018) is one of the rare studies that did not perform feature engineering, but applied a 3DConvLSTM model on full 4D fMRI data. They obtained an accuracy of 73% on a relatively small dataset of 50 subjects.

### 3.5 Effect of sample size and number of sites

**Fig. 5:**
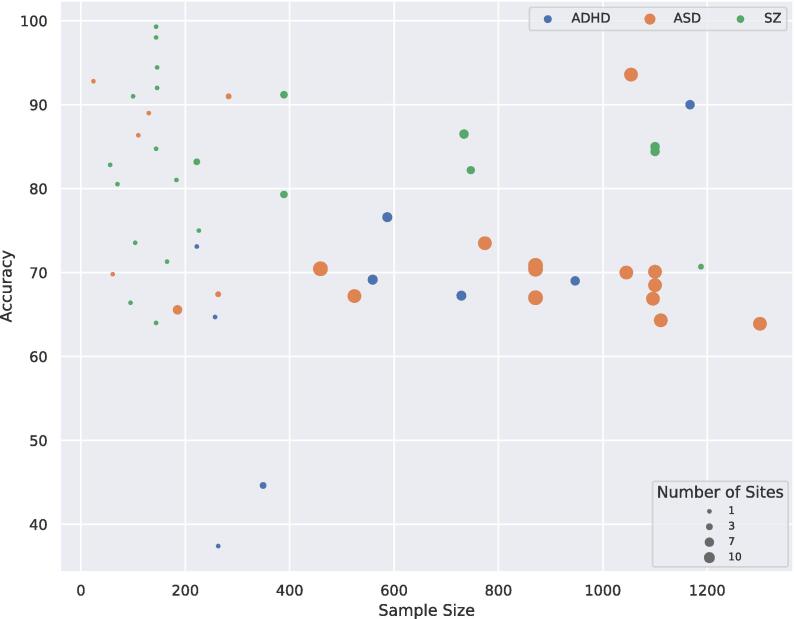
Scatterplot of accuracy for different sample sizes, the size of the dots indicates the number of scanning sites included in the sample.

The effect of sample size on accuracy is illustrated in Fig. 5. Although there is no obvious linear relation, there is a significant negative monotonic relation between sample size and accuracy when combining all the studies (*r_s_ =* -0.32, *p* = 0.02). Though when splitting the data per disorder, these trends did not reach significance and were even absent or in opposite direction (ASD: *r_s_ =* -0.42, *p* = 0.05; SZ: *r_s_ =* 0.02, *p* = 0.94; ADHD: *r_s_ =* 0.43, *p* = 0.24). When splitting the data for number of sites, no significant relation was observed (see Fig S1, S2).

We repeated the correlation analysis between sample size and accuracy after excluding nine ASD or SZ studies where feature selection on the entire sample cannot be ruled out (Li G. et al., 2018, Wang et al., 2019, Kam et al., 2017, Yan et al., 2019, Qureshi et al., 2019b, Qureshi et al., 2017, Vyskovsky et al., 2019) or where cross-validation was lacking while working on a small sample (n < 50) (Ismail et al., 2017). These results also showed a significant negative relation between sample size and accuracy on the full dataset *(*r_s_ = *-0.42,* p *= 0.002).* When splitting the data per disorder, this trend was only significant for ASD *(ASD:* r_s_ = *-0.51,* p *= 0.03; SZ:* r_s_ = *0.15,* p *= 0.57; ADHD:* r_s_ = *0.43,* p *= 0.24).*

Naturally, larger samples usually involve more scanning sites, thus more heterogeneity in the data. It also shows that SZ studies have more studies with high performances (>90% accuracy), but that most of these are conducted on small datasets. ASD studies often involve large sample sizes with many scanning sites, which could be explained by the publicly available ABIDE dataset.

### 3.6 Deep learning vs. Standard machine learning

**Fig. 6:**
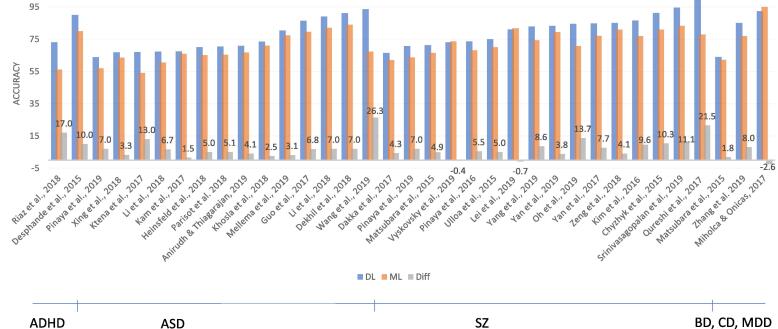
Results of studies comparing DL and conventional ML models. The graph shows the accuracies (or other reported performance scores: AUC, balanced Acc, F score) for DL models in blue and ML models in orange. The difference between the two groups is depicted in grey. (For interpretation of the references to colour in this figure legend, the reader is referred to the web version of this article.)

A total of thirty-five studies included in this review compared a DL model against a standard machine learning method (such as SVM, LR or RF). The results of these studies are shown in Fig. 6. For thirty-two of the thirty-five included studies (91%), DL showed improved performance as compared to SML. Given the heterogeneity of the input of the models, it is difficult to identify specific characteristics of the studies associated with greater improvement when applying DL. The difference seems to go up whenever DL models are gaining higher performances. Only three studies report lower performance for DL than SML (Lei et al., 2020, Miholca and Onicaş, 2017, Vyskovsky et al., 2019): Lei et al. (2020) compared many different models of which SVM achieved the highest performance on the AAL FC matrix. The 2D convolutional neural network only performed slightly worse (difference of 0.7%). In Vyskovsky et al. (2019) an ensemble of MLPs was outperformed by an ensemble of SVMs for first episode schizophrenia classification with a marginal difference of 0.4%. Finally, in Miholca and Onicaş (2017) a new kind of ML technique using relational association rules achieved a 2.6% better accuracy score than an MLP.

## 4 Quantitative *meta*-analysis

**Fig. 7:**
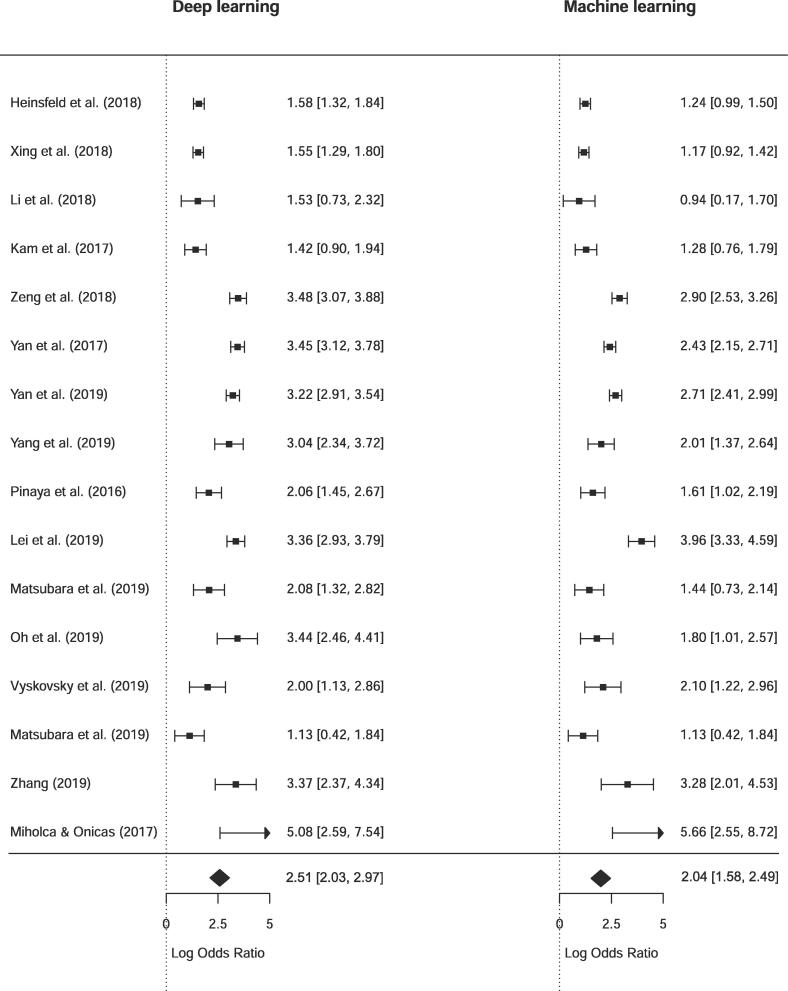
Forest plot of diagnostic odds ratio for deep learning and machine learning comparison.

To test whether DL techniques achieved significantly higher performances than SML techniques, we performed a quantitative *meta*-analysis for 16 studies that 1) directly compared a DL model with SML and 2) reported sensitivity and specificity results to perform *meta*-analysis for bivariate classification. Fig. 7 shows an illustrative forest plot of the included studies. The pooled DOR for deep learning models was 2.51 [2.03, 2.97] versus 2.04 [1.58, 2.49] for standard machine learning models. To test whether this difference was significant we performed a random-effect *meta*-regression for type of model, for which the results are presented in Table 5. Although DL had a higher odds ratio, the difference between the two estimates was not significant (*p = 0.165*). When comparing sensitivity and false positive rates (fpr) separately according to the Reitsma model, DL had a higher sensitivity, but the difference was again non-significant (*p = 0.779*). The false positive rates were higher for machine learning models (*p = 0.032*), but this did not remain significant after Bonferroni correction for multiple comparisons.

**Table 5:** bivariate random-effect *meta*-regression with DL/ML as covariate

	Point Estimate	SE	z value	p value	95% CI lower	95% CI upper
All studies (n = 16)						
DOR (*higher is better)*	0.464	0.334	1.387	0.165	−0.192	1.119
Sens (*higher is better)*	0.068	0.242	0.281	0.779	−0.406	0.542
Fpr *(lower is better)*	−0.419	0.195	2.149	0.032*	−0.801	−0.037
ASD (n = 4)						
DOR (*higher is better)*	0.347	0.128	2.721	0.007**	0.097	0.597
Sens (*higher is better)*	0.181	0.203	0.888	0.374	−0.218	0.579
Fpr *(lower is better)*	−0.162	0.183	−0.884	0.377	−0.520	0.197
SZ (n = 9)						
DOR (*higher is better)*	0.601	0.331	1.814	0.070	−0.048	1.250
Sens (*higher is better)*	0.086	0.328	0.261	0.794	−0.558	0.729
Fpr *(lower is better)*	−0.519	0.217	−2.396	0.017*	−0.944	−0.095

Bivariate random effects meta regression results with DL/ML as covariate. Results are indicated as estimates for DL, thus a higher point estimate for sensitivity indicates higher sensitivity for DL results as compared to ML.

* Significant at the 0.05 level without Bonferroni correction

** Significant at the 0.05 level with Bonferroni correction

When pooling studies that investigated the same disorder, there was only a significant increase in DL performance in ASD (n = 4) as measured by increased odds ratio (*p = 0.007*). For SZ (n = 9), there was only a significant difference for false positive rate (*p = 0.017*) with SML results showing higher fpr, but this did also not remain significant after Bonferroni correction for multiple comparisons.

### 4.1 Pooled DOR per disorder

**Fig. 8:**
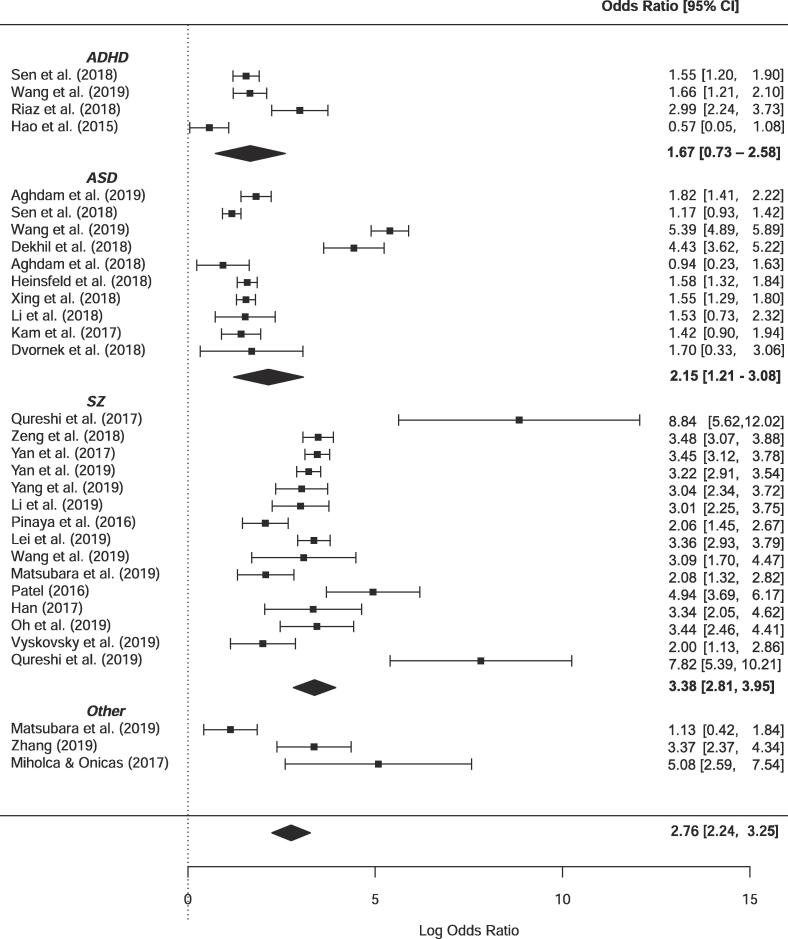
Univariate random-effect forest plots of log diagnostic odds ratio’s grouped per disorder.

The univariate forest plot of DOR of all studies included in the *meta*-analysis is shown in Fig. 8. The total pooled DOR of DL studies was 2.76 [95% CI = 2.24–3.25]. Pooled DOR for ADHD studies was lowest with 1.67 [95% CI = 0.73–2.58], followed by ASD with a pooled DOR of 2.15 [95% CI = 1.21–3.08] and the highest for SZ studies with a pooled DOR of 3.38 [95% CI = 2.81–3.95]. Again, it can be seen that there is large variety in performance of models within a disorder, which is probably caused by sample variance as inter-study differences are present in population, modalities, type of DL model, feature selection and engineering technique.

## 5 Discussion

### 5.1 General conclusions from the existing literature

In the present review we systematically reviewed the literature applying deep learning methods to neuroimaging data for psychiatric disorders. Despite many promising results, the clinical use of DL on neuroimaging data to aid disease diagnosis for psychiatric disorders is still in its infancy. Given the complexity of the problem, starting from inherently uncertain diagnostic labels to heterogeneous scanning protocols and preprocessing, this is perhaps not surprising. Nevertheless, in recent years many studies have applied DL techniques to classify psychiatric disorders. While the body of literature on ASD, SZ or ADHD is increasing steadily, only a few studies have applied DL on other disorders such as MDD. It seems that the large, publicly available datasets are driving research as many of the included studies were based on ABIDE, COBRE and ADHD-200 datasets. Furthermore, the way that these datasets provide the neuroimaging data seems to influence what kind of features have been used as input for DL. For example, the ABIDE offers preprocessed timecourses for various atlas parcellations and many ASD studies use atlas extracted timecourses or FC matrices as input. In contrast, in SZ studies the input is highly heterogeneous. Even though multiple studies are using the same datasets, it remains difficult to compare performances and to identify optimal models or feature input. Various studies still use different subsets of the available dataset due to different quality checks or preferences. Furthermore, due to the rapid development of DL techniques and the wealth of preprocessing and parameter choices, there is large heterogeneity in models used and features engineered.

Only a few studies have directly compared either differently engineered features or different modality approaches, making a definite conclusion on specific inputs difficult. Resting-state fMRI seems to be used most often, but whenever structural MRI is used, it achieves similar accuracies. The vast majority of studies apply a form of feature engineering to the data, instead of developing end-to-end models for MRI that could learn features from the raw data.

From the three main disorders discussed, SZ seems to obtain the highest classification performance. There are several non-exclusive possibilities that may explain the differences in performance. One possibility is that the labels for SZ are more reliable. However, the inter rater reliability (IRR) for SZ appears lower than for ASD and ADHD (Regier et al., 2013). Yet, it is important to note that also IRR of these different disorders is difficult to compare as they have been assessed in different settings. (Regier et al., 2013) Another possibility is that the samples were more homogenous. Fig. 5 indicates that the samples for SZ were smaller and obtained at fewer sites. This could have reduced the clinical heterogeneity within the patient group that is associated with higher accuracy (Schnack and Kahn, 2016), as well as the heterogeneity of the imaging data. This is also consistent with the observation that accuracy was higher with smaller sample sizes (Fig. 5), which is in line with reviews for standard ML (Woo et al., 2017, Wolfers et al., 2015). However, this pattern was absent for SZ and even positive for ADHD, suggesting that the overall negative association was primarily driven by the negative trend for ASD studies. Another explanation may be that differences in brain anatomy and function are more distinct from healthy controls. This is supported by data from large-scale neuroimaging consortia that have reported larger differences in brain anatomy for SZ compared with ADHD and ASD (Thompson et al., 2020).

Remarkably, only half of the studies report sensitivity or specificity performance measures, whereas these are important for interpretation, especially when case/control groups have unequal sample sizes (Scheinost et al., 2019), and these measures are required for the present *meta*-analysis based on diagnostic odds ratios. Furthermore, many studies do not compare their model with a benchmark SML model. This hinders a comprehensive comparison and a quantitative analysis of all included studies. In the following section we will evaluate more good and bad practices.

### 5.2 Good and bad practices

In general, it can be concluded that there are still a considerable number of studies not adhering to the ten simple rules of individual differences in neuroimaging as proposed by Scheinost et al. (2019). We will discuss four themes based on these ten rules and our findings from this review:

#### 5.2.1 Out-of-sample predictions

The first rules of Scheinost et al. (2019) describe the need for an out-of-sample prediction as it generates more accurate and generalizable models. Predictive models in neuroimaging can be susceptible to overfitting, the tendency to mistakenly fit sample-specific noise as if it were signal (Yarkoni and Westfall, 2017), especially since the number of predictors is usually far greater than the number of observations (Whelan and Garavan, 2014). Common practice to deal with the curse of dimensionality in neuroimaging is feature selection or engineering. This should be done carefully as training and test set need to stay independent. In our reported tables the highest reported accuracies are often from studies doing feature selection and we need to carefully interpret these results. Doing feature selection is not a bad practice, but it should be done inside a cross validation loop or on a different dataset (Scheinost et al., 2019). At least for three studies (Li G. et al., 2018, Yan, 2017, Yan et al., 2019) feature selection is done on the whole sample, leading to model ‘peeking’ into the test data, which may lead to optimistic results. For several other studies it was unclear whether this procedure was done properly (Wang et al., 2019, Qureshi et al., 2019b, Qureshi et al., 2017).

#### 5.2.2 Proper cross-validation

As discussed in rule number 3 of Scheinost et al. (2019), cross validation should be used to test a model’s generalizability. Preferably even, the model should be tested on a separate, external dataset as this provides most evidence of model generalization, but this is often not feasible. Still, several studies only report accuracies based on a single train/test split (Li G. et al., 2018, Srinivasagopalan et al., 2019, Kuang et al., 2014, Kuang and He, 2014, Li et al., 2019), therefore reporting an overly optimistic outcome and complicating comparisons with other studies. As the best practice for model generalizability is to use an independently collected dataset as test set, it is good practice to report leave site out validation as each site is an independent dataset. This is not yet standard practice as many studies have used multi-site data, but only few report leave-site-out cross-validation.

#### 5.2.3 Choice of model and performance metric

The choice of model and performance metric should be defined prior to the analysis and heavily depends on the question of interest. Questions of interest about comparisons of SML and DL models should be carefully designed and define models and methods of parameter optimization before analysis. It is important to note that using pre-engineered features for SML and DL models can lead to an unfavourable comparison for DL as its main advantage is representation learning. Instead of focusing on the highest performance score, questions of interest could also focus on exploring the possibilities of DL models on minimally preprocessed data, as preprocessing involves many subjective choices. In this review we conclude that there is both a lack of proper comparisons between SML and DL models on the same, pre-engineered input features as well as studies of DL models that explore the possibilities of DL applications to higher dimensional data such as 3D or 4D images.

Finally, when the ultimate goal is to understand the relationship between behaviour and brain activity, the interpretation of results matters (Scheinost et al., 2019). If a complex model yields better performance but is less able to map the brain-behaviour associations, simpler models may be preferred. In this review several studies try to map the findings of DL models to specific brain areas (Sen et al., 2018, Matsubara et al., 2019, Xing et al., 2018, Yan et al., 2019, Kim et al., 2016, Deshpande et al., 2015, Vyskovsky et al., 2019, Castro et al., 2015, Pinaya et al., 2016), but there is still a lack of comparisons between those highlighted brain areas across studies or between DL and SML models.

In conclusion, different questions of interest ask for different approaches and should therefore be defined properly and prior to the analysis.

#### 5.2.4 Reporting statistics and code

Moreover, not only accuracy should be reported, as overall accuracy may not translate well to accuracy for individual classes (Baldi et al., 2000). Studies should at least also report sensitivity and specificity. Furthermore, when comparing models’ performance, it is crucial to perform statistical analysis of performance gains before drawing any conclusions. Statistical significance is best evaluated using permutation testing, since results of each fold of the cross-validation are not independent, or with external validation on an independent dataset (Scheinost et al., 2019). Finally, although a considerable number of studies already shares data and code, this should become standard practice to facilitate external validation and model comparisons.

### 5.3 Deep learning vs machine learning

Although DL has unlocked unprecedented success in various domains, its superiority as an analytical tool for neuroimaging in psychiatry is yet to be demonstrated. The added benefit of DL is its ability to capture nonlinear, subtle patterns, but the question arises whether these nonlinearities 1) exist between brain connectivity and psychiatric disorders and 2) are exploitable at the currently available sample sizes and examined scales. In this review we tried to examine the difference in performance between DL and standard, shallow ML models in the classification of psychiatric disorders. As can be seen in Fig. 6, for thirty-two out of thirty-five studies (91%) directly comparing DL to SML, the performance of DL models was higher. When statistically comparing the two techniques on the sixteen studies that did report sensitivity and specificity, which is necessary for *meta*-analysis on odds ratios (Macaskill et al., 2010), no significant difference was obtained. This could merely be the result of insufficient power, or because the random-effect *meta*-regression with SML/DL covariate assumes that the data arise from a randomized design. This is a conservative approach as the results truly are paired results; they are obtained by application of both techniques to the same dataset. Unfortunately, there is a lack of *meta*-analytical models that account for pairing of test results (Macaskill et al., 2010) and we can therefore not apply a more appropriate and possibly more liberal approach. We assume that a paired analysis will show significant better performance of DL techniques as DL performed better in 91,43%% of the included studies that compare both methods, and we have seen that comparisons of SML-DL within one disorder does lead to significant differences.

The outperformance of DL compared to SML may be partly explained by a publication bias given the increased interest in DL and our search for DL papers specifically (Boulesteix et al., 2013). It is, for instance, likely that many included studies optimized parameters for their DL model but did not optimize parameters for their comparative SML model. The difference with and without optimisation can be large: In a study of (Yang et al., 2019, Yang et al., 2019) a grid search method was deployed to find the optimal parameters for SVM. They obtained a cross validated accuracy of 71.98% on the entire ABIDE I, whereas without optimisation (Heinsfeld et al., 2018) report an accuracy of 65% using SVM on the ABIDE I. It is therefore important to have standardized procedures for fair comparison between DL and SML models, where the models and methods of parameter optimization are chosen beforehand. Furthermore, studies should test whether the difference in performance is significant to properly benchmark the potential added value of DL models.

The overwhelming outperformance of DL studies is still surprising given that the majority of studies used pre-engineered features for classification, whereas the main advantage of DL comes from learning non-linearities of minimally preprocessed data (Abrol et al., 2020). A recent study suggested that DL is better able to fit brain connectivity, even when the data is preprocessed (using connectivity features of 400 regions). They showed that DL performs particularly well at connector hubs - regions with diverse connectivity (Bertolero and Bassett, 2020). It is still unclear whether the discussed models of this review have also picked up these non-linearities or whether there are more specific cases where DL could be particularly beneficial. We do know that only a few studies have exploited DL’s capabilities of representation learning, meaning that the ‘true’ value of DL performance still remains to be deciphered.

### 5.4 Strengths and limitations

We will shortly discuss the strengths and limitations of this review and *meta*-analysis. First of all, given the high interest in DL and rapid increase of DL studies in neuroimaging, there was a need for a systematic overview of DL applications in psychiatry. Given the rigorous search in technical and biologically oriented databases, we included a large amount of studies in an attempt to give a comprehensive overview. One important limitations of this overview is the lack of an extensive quality assessment of studies as is proposed by the Cochrane handbook (Macaskill et al., 2010). This may have led to inclusion of studies of less quality and biased results. However, this enabled us to identify good and bad practices within the field. Furthermore, for a good comparison between SML and DL studies, a thorough investigation on publication bias is needed to establish the reliability of this trend in favour of DL.

The most important limitation for the *meta*-analysis is that we could only include a small amount of studies for quantitative analysis as most studies did not report sensitivity or specificity performances. Whenever more studies can be included, this would aid the generalization of our conclusions. An important boost for statistical power would be to include AD studies in this *meta*-analysis as numerous studies have applied DL models for AD classification (Jo et al., 2019, Vieira et al., 2019). Although the identification of AD is usually based on structural rather than functional neuroimaging, the inclusion of AD studies in a future *meta*-analysis may enable the identification of a reliable baseline to validate future studies. Finally, performing a paired *meta*-regression would aid in the comparison of DL-SML performances, but appropriate methods for doing so still need to be developed.

## 6 Conclusions and future directions

Effective and accurate diagnosis of psychiatric disorders is important for initiation and choice of effective treatment. This review confirms that DL on neuroimaging is a promising tool for development of biological diagnostic models that could aid diagnosis. While still in its early stages, the application of DL in neuroimaging for psychiatric disorders has shown promising results and obtained better performance than conventional shallow machine learning techniques. Nevertheless, several improvements are needed before the full potential of DL in psychiatric neuroimaging can be achieved. The fifty-five studies included in this review show a wide variety of patient characteristics, type of feature engineering and applied DL techniques which raises problems of generalizability. Due to these heterogeneous approaches, we could not identify optimal models or approaches for bivariate classifications.

When choosing a model and reporting its accuracy, future studies should be mindful of the questions of interest they want to answer. If the aim is to develop a new DL model to improve performance, an extensive, neutral comparison to benchmarked SML models should be made that includes important performance measures for diagnostic classification (including sensitivity and specificity). Alternatively, the aim could be to apply DL to different kinds of input data, as it can learn features from higher dimensional data than SML techniques. Yet, we have seen that many studies still use linear feature engineered inputs, suggesting that the DL models are not used to their full potential. In general, studies should report extensive performance comparisons and keep in mind the ten rules for predictive modelling of individual differences (Scheinost et al., 2019) including proper validation.

Since we found that publicly available datasets drive research, we suggest that our recommendations are best implemented bottom-up, by introducing standardized datasets, with standardized preprocessing protocols. Ideally, all code for models using these datasets should be publicly available. Similarly, not only the performance results should be reported, but the full data of (in)correct classification of all subjects should be made available to make a proper comparison between models. This would also help to identify subject IDs that are always classified wrong, which could aid to identify noise in the diagnostic labels.

In conclusion, neuroimaging research in psychiatry using deep learning is still evolving to achieve better performance. While there are important challenges to overcome, our findings provide preliminary evidence supporting the promising role of DL in the future development of biological neuroimaging biomarkers for psychiatric disorders.
