# Supplementary figures and images for: Deep learning applications for the classification of psychiatric disorders using neuroimaging data: Systematic review and meta-analysis
Source: Neuroimage Clin. 2021 Feb 10;30:102584. doi: 10.1016/j.nicl.2021.102584 (PMC8209481; doi:10.1016/j.nicl.2021.102584)

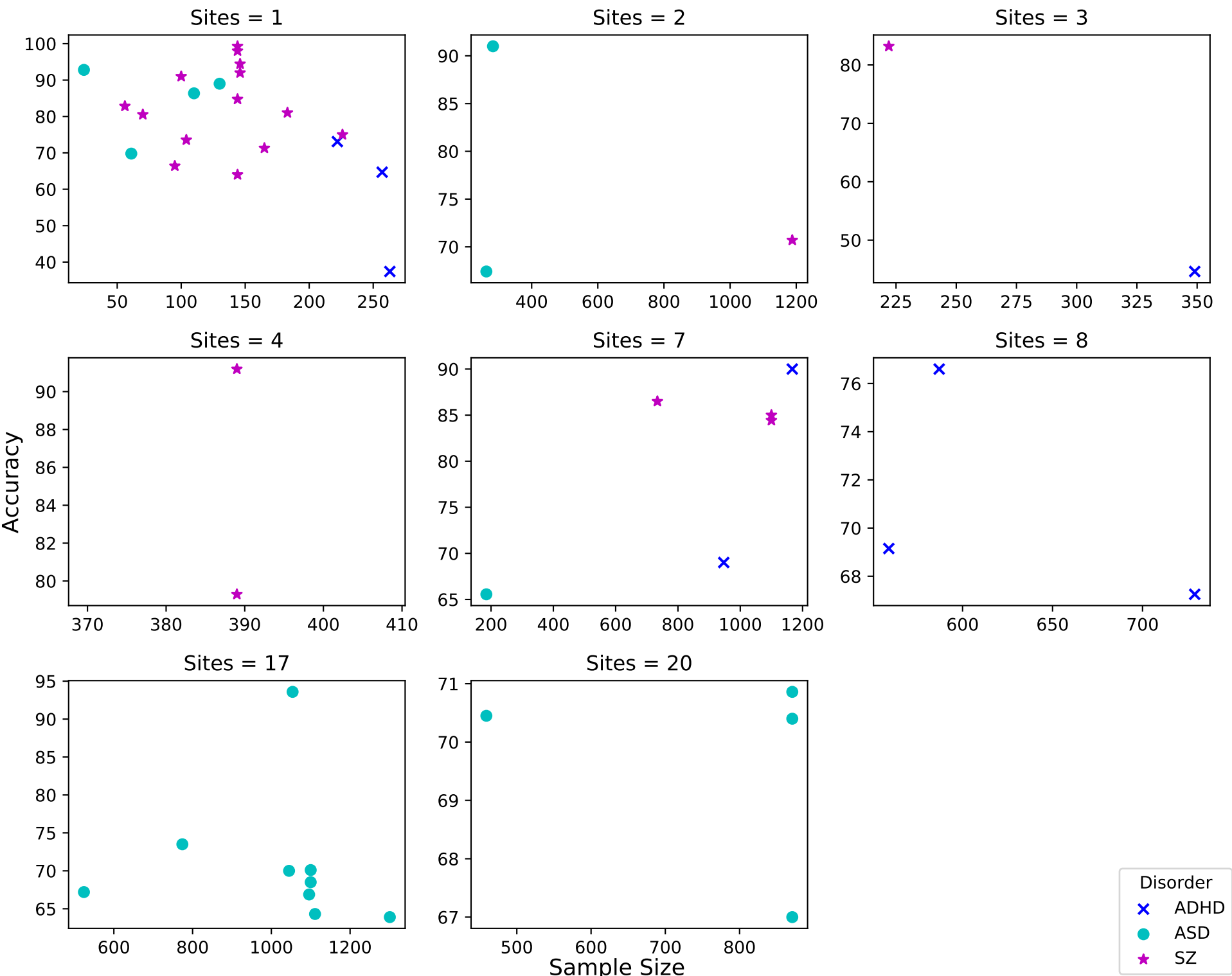

Supplement: Supplementary data 1 [file mmc1.pdf]

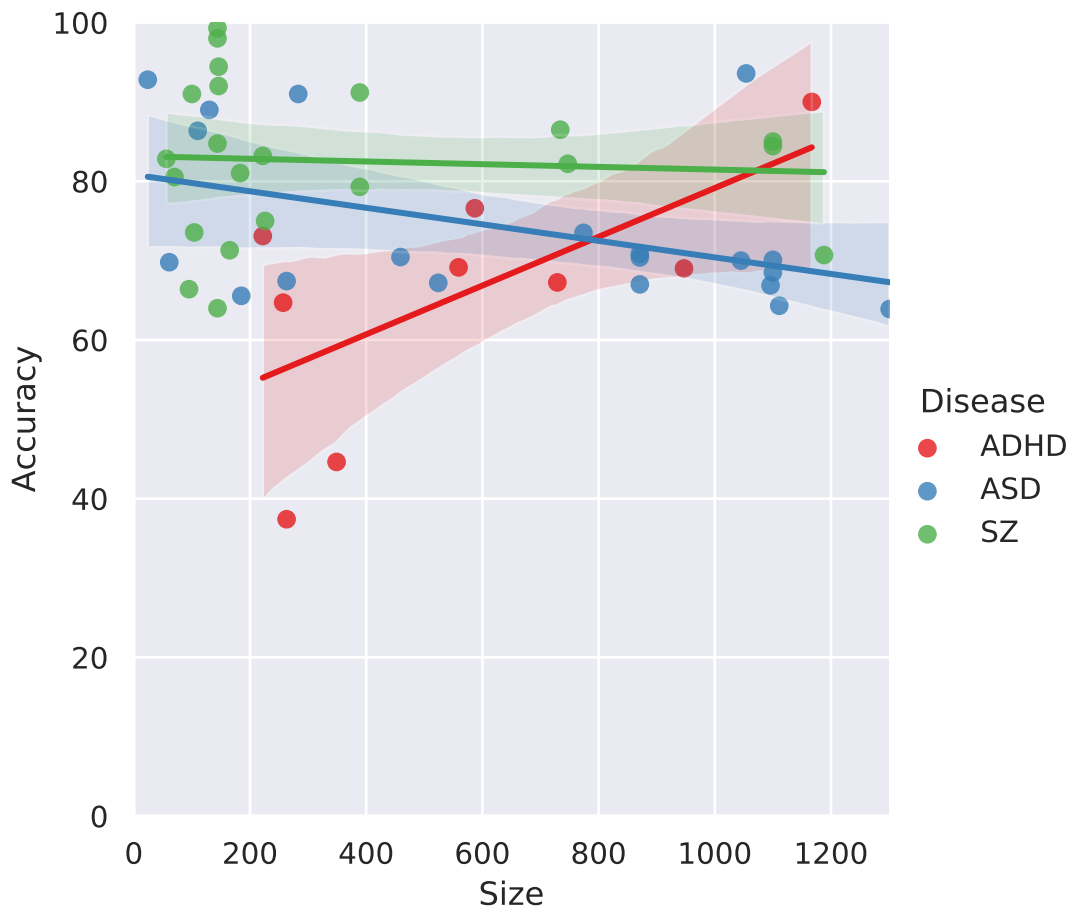

Supplement: Supplementary data 2 [file mmc2.pdf]
